# Application research on the quantitative evaluation of carotid arterial elasticity in overweight adolescents using ultra-fast pulse wave technology

**DOI:** 10.3389/fendo.2026.1619692

**Published:** 2026-03-03

**Authors:** Qiaoer Gong, Xueli Zhu, Nianyu Xue

**Affiliations:** Department of Ultrasound, The First Affiliated Hospital of Ningbo University, Ningbo, China

**Keywords:** adolescents, carotid artery elasticity, overweight, ultrafast pulse wave velocity, ultrasonography

## Abstract

**Background:**

Overweight and obesity in adolescents has become a new worldwide health problem. Overweight in adolescence not only leads to persistent overweight and even obesity in adulthood, but also leads to decreased arterial function in adolescence and a greatly increased incidence of chronic diseases in adulthood. However, current imaging techniques cannot detect the early decrease of arterial elasticity. In this study, Ultrafast pulse wave velocity (UFPWV) technology was used to quantitatively evaluate the carotid artery elasticity in adolescents with simple overweight. In order to find the changes of carotid artery elasticity and related influencing factors in overweight adolescents at an early stage.

**Data and methods:**

A total of 56 adolescents who met the inclusion and exclusion criteria were enrolled in this study, including 30 adolescents with Body mass index (BMI) ≥24kg/m^2^ as overweight group and 26 adolescents with normal clinical signs and examinations as normal group. Clinical data and biochemical parameters were collected and analyzed for all participants, along with measurements of carotid intima-media thickness (IMT) and carotid arterial elasticity, including pulse wave velocity at the beginning of systole (PWVBS) and at the end of systole (PWVES).

**Results:**

The mean values of IMT, PWVBS and PWVES in overweight group were higher than those in normal group. There was significant difference between PWVBS and PWVES (*P*< 0.05), but not IMT (*P >*0.05). PWVBS and PWVES in overweight group were positively correlated with BMI, uric acid (UA), total cholesterol (TC) and low-density lipoprotein cholesterol (LDL-C) (all *P*< 0.05). And negatively correlated with High-density lipoprotein cholesterol (HDL-C) (*P*< 0.05). Within the overweight group, the subgroup with elevated uric acid (UA) levels showed significantly higher arterial elasticity parameters compared to the subgroup with normal UA levels (*P* < 0.05), whereas no statistically significant difference was observed in IMT between the two subgroups (*P* > 0.05). Receiver operator characteristic curve (ROC) analysis showed that when PWVES>4.515 m/s was used as the cut-off value of abnormal carotid elasticity in overweight adolescents in this study, the sensitivity was 71.8%. The specificity was 73.2%.

**Conclusion:**

UFPWV can detect early changes in carotid artery elasticity in overweight adolescents. When hyperlipidemia and hyperuricemia coexist, they exert a synergistic detrimental effect on arterial elastic function. The arterial elasticity indexes PWVBS and PWVES are significantly correlated with a variety of traditional risk factors of atherosclerosis (AS), which can be used AS effective indicators to predict early AS.

## 1 Introduction

Since 1980, the obesity rate among children in over 70 countries has more than doubled, particularly in some developing nations where the increase has been even greater, with rates of childhood obesity rising significantly faster than those of adults (1). In 2015, 107.7 million children were classified as obese globally. The World Obesity Federation estimated in 2019 (2) that by 2020, there would be 158 million children and adolescents aged 5 to 19 years suffering from obesity worldwide, with projections of 206 million by 2025 and 254 million by 2030. By 2030, China is expected to be one of the countries with the most significant obesity problems, with over one million obese adolescents in 42 countries (3).

Obesity and being overweight can be categorized based on their causes (4): simple (primary) and symptomatic (secondary), with simple obesity accounting for up to 95% of cases. Despite this prevalence, managing simple obesity poses considerable challenges, requiring greater attention. Overweight in adolescents not only exacerbates psychological burdens, leading to feelings of inferiority, anxiety, and depression, but also induces pathological changes. Dyslipidemia and hypercholesterolemia resulting from being overweight are major risk factors for AS. Research has shown (5) that the pathological process of AS begins not only in adulthood but early in overweight adolescents. Evidence of AS has been found in the coronary and abdominal aortas of infants and adolescents who did not exhibit cardiovascular diseases (CVD) at autopsy. Follow-up of overweight adolescents found that they also had substantially increased odds of CVD in adulthood (6). However, another study indicates that the functional changes caused by AS precede morphological changes (7, 8). Currently, most imaging techniques focus on detecting morphological changes in arteries, highlighting the necessity of early monitoring of vascular elasticity function in adolescents to prevent the onset of CVD in adulthood.

Currently, a well-established system exists for assessing the carotid artery. As a superficial and readily accessible vessel, the carotid artery is ideally suited for ultrasound examination, which offers real-time imaging, non-invasiveness, high reproducibility, and low cost. Consequently, carotid IMT is widely regarded as a surrogate marker of vascular complications, capable of detecting early atherosclerosis and serving as a predictive indicator for future myocardial infarction and stroke (9). However, previous studies (7, 8) have indicated that functional changes in blood vessels occur before morphological changes, rendering IMT less suitable for monitoring early arterial changes in overweight adolescents. Greater emphasis should be placed on assessing changes in vascular functional elasticity. Several conventional ultrasound-based techniques—including vascular echo-tracking, wave intensity analysis, real-time shear wave elastography (SWE), and the cardio-ankle vascular index (CAVI)—have been used to evaluate arterial elasticity. However, each has notable limitations: vascular echo-tracking is highly susceptible to respiratory motion, resulting in unstable measurements. Wave intensity analysis primarily reflects cardio-vascular interactions and requires costly equipment and complex procedures, limiting its utility in routine clinical practice. SWE is predominantly used for assessing elasticity in organs like the liver, breast, and thyroid—tissues minimally affected by cardiac or respiratory motion—and is rarely applied to vascular tissue. Furthermore, there is no unified standard, and it also relies on the operator’s technique. CAVI estimates overall arterial stiffness along a long vascular path from the aortic root to the ankle artery, this extended trajectory increases susceptibility to measurement error, reducing accuracy.

Among various methods for functional arterial monitoring, pulse wave velocity (PWV) is considered the non-invasive “gold standard” for assessing arterial stiffness and has been closely linked to risks of AS and CVD mortality (10). Its measurement has been included in the European Guidelines on Hypertension Prevention and Treatment since 2003. However, this technique suffers from complexities in operation and poor distance stability, with average measurements of the tested vascular segment failing to accurately reflect local arterial conditions. Additionally, PWV measurements can be affected by pressure wave reflections and overlays, leading to inaccuracies. In response, researchers have introduced a new vascular imaging technology known as UFPWV technology, which achieves precise quantification of vascular elasticity function through high-frequency imaging at up to 20,000 frames per second. This technique has already been successfully applied to assess arterial elasticity in multiple clinical contexts, including diabetic vasculopathy (11), hypertensive arteriosclerosis (12), vascular evaluation in rheumatic diseases (13), and vascular injury associated with sleep-disordered breathing (14). Moreover, it has been recognized as an important detection indicator for early warning and intervention of CVD (15). Compared to traditional methods, UFPWV offers several advantages (16): it can directly measure PWV values at specific vascular points, enhancing detection accuracy; the operational process is simplified, with minimal external interference; and it requires no additional equipment for offline calculations. A literature review reveals that UFPWV has primarily been applied in studies of carotid artery elasticity in adults, with no reported studies focusing on overweight adolescents. Therefore, this research aims to apply UFPWV technology for the quantitative analysis of carotid elasticity in adolescents with simple overweight and to explore the impact of various biochemical indicators on vascular elasticity.

## 2 Methods

### 2.1 Case selection and grouping

This study selected 56 adolescents who visited Ningbo University First Affiliated Hospital from March 2023 to December 2024. Based on the “Classification Standards for Obesity and Obesity Screening Weight Index Values in Chinese School-age Adolescents” (17), subjects with a BMI ≥24 kg/m^2^ were classified into the overweight group, while those with a BMI<24 kg/m^2^ were classified into the normal group.

(1) Overweight Group: 89 overweight adolescents were initially enrolled.

Inclusion criteria:

1. Age ≤ 18 years;
2. Met the diagnostic criterion for overweight: BMI ≥ 24 kg/m^2^.

Exclusion criteria:

1. Poor cooperation during examination or inadequate ultrasound image quality (n = 38);
2. Incomplete clinical data (n = 29);
3. Secondary obesity due to diabetes, hypertension, or other systemic diseases (n = 12);
4. History of smoking or alcohol use (n = 5);
5. Coefficient of variation Δ± > 20% between repeated UFPWV measurements of PWVBS and PWVES (n = 16);
6. Presence of carotid plaques or increased common carotid artery IMT on B-mode ultrasound (n = 12).

(2) Control Group: 42 healthy adolescents were randomly selected during the same period from routine health check-ups at the First Affiliated Hospital of Ningbo University.

Inclusion criteria:

1. Age ≤ 18 years;
2. Normal weight defined as BMI < 24 kg/m^2^.

Exclusion criteria:

1. Diagnosis of diabetes, hypertension, or other cardiovascular or cerebrovascular diseases (n = 13);
2. History of smoking or alcohol use (n = 1);
3. Any abnormality in routine blood/urine tests, liver or renal function, fasting glucose, electrocardiogram, or abdominal ultrasound (n = 12).

Final cohort:

Overweight group: 30 adolescents (age range: 10–17 years; mean age: 15 years);

Control group: 26 adolescents (age range: 8–17 years; mean age: 13 years).

This retrospective study was approved by the Ethical Committee of Ningbo University First Affiliated Hospital (Approval No. 053RS of the 2025 Ethics Review).

### 2.2 Instruments and equipment

The study utilized the Supersonic Imagine Aixplorer^®^ color Doppler ultrasound diagnostic system, with the probe model SL10–2 linear array probe operating at a frequency range of 2–10 MHz. The system features an integrated UFPWV detection module, and the preset mode was configured for carotid artery assessment.

### 2.3 Measurement indicators and methods

(1) Collection of General Clinical Data.

All participants’ clinical data were collected and recorded, including age, height, and weight.

(2) Collection of Biochemical Test Results.

Participants were instructed to have a light diet the night before blood sampling, fasting for more than 12 hours. Blood was drawn the following morning to obtain fasting plasma glucose(FPG),UA, TG, TC, HDL-C, and LDL-C.

(3) Measurement of IMT.

**Figure 1:**
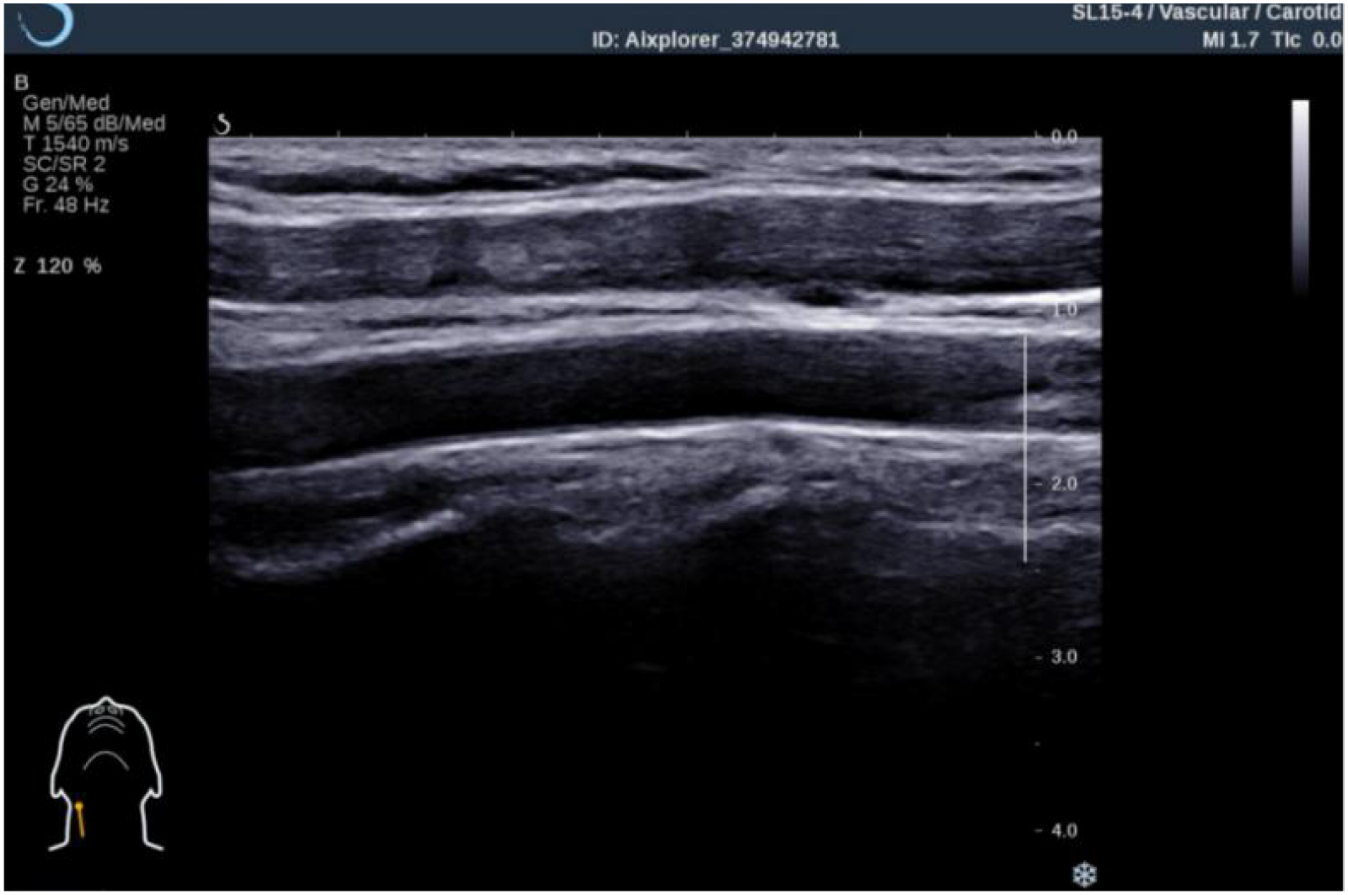
Long-axis image of common carotid artery under two-dimensional gray-scale ultrasound.

Participants were positioned supine with their necks extended and turned toward the opposite side to adequately expose the carotid artery. The ultrasound probe was placed 1.5 cm proximal to the bifurcation of the common carotid artery. After obtaining a clear longitudinal image, the image was frozen (**Figure 1**). The built-in IMT measurement function of the instrument was activated, and the system automatically calculated and recorded the average IMT value for that vascular segment.

**Figure 2:**
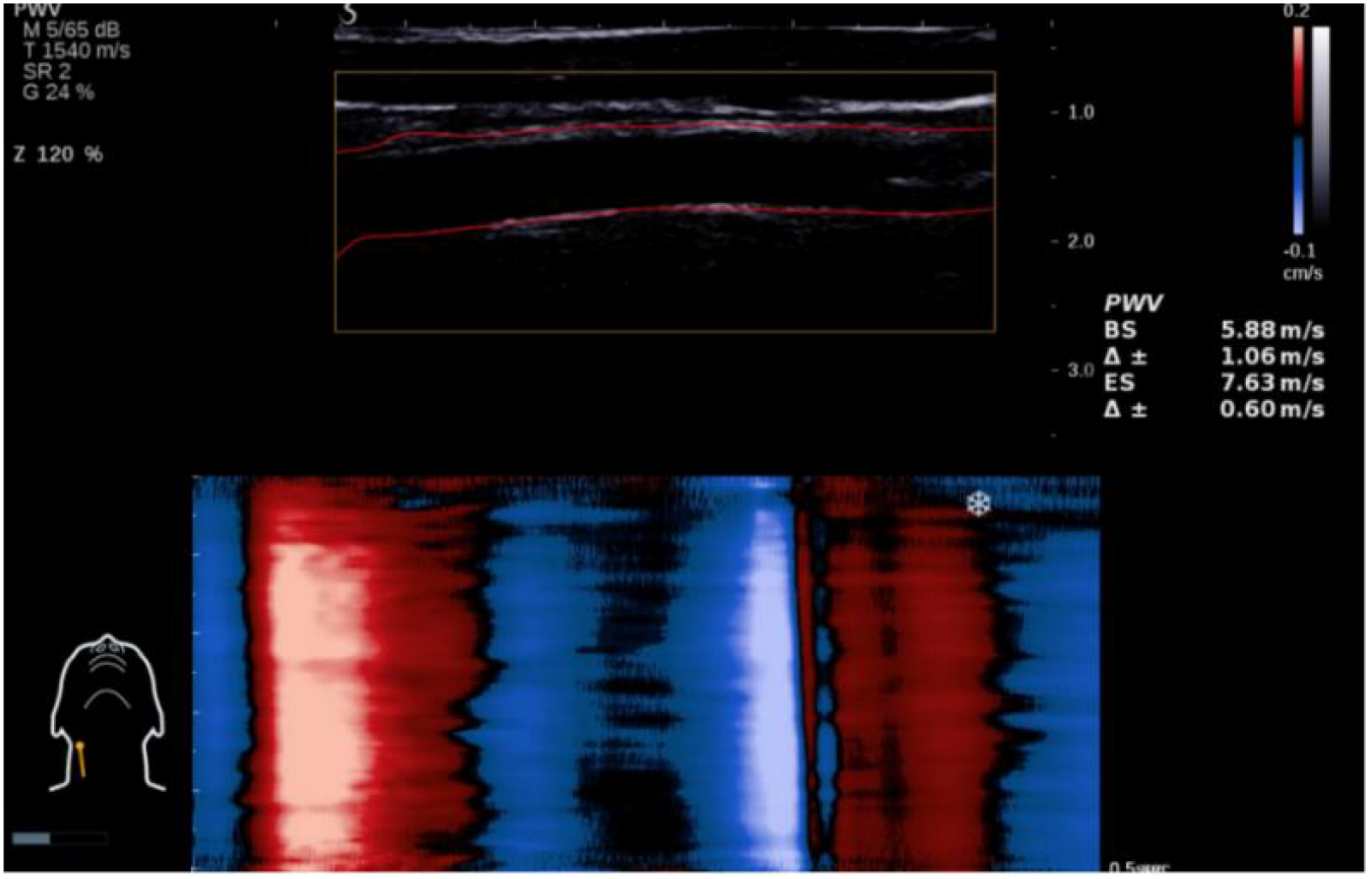
Measurement of UFPWV in the anterior wall of the carotid artery.

(4) UFPWV Measurement:

Participants were instructed to remain quiet and hold their breath for 5 seconds. The operator clicked the “PWV” button on the control panel and maintained the probe’s stability for approximately 5 seconds. Once the system automatically identified the endothelial line of the blood vessel, the operator clicked the “Select” button on the control panel to obtain data for PWV at the beginning of systole (PWVBS) and at the end of systole (PWVES), along with their coefficients of variation (Δ±). Ideally, Δ± should be less than 20% (**Figure 2**). Each participant underwent three repeated measurements, and the final result was taken as the average value. All procedures were independently performed and verified by two ultrasound physicians with over five years of experience.

In the accompanying **Figure 2**, the yellow box indicates the adjustable sample frame for the region of interest, while the red dashed line represents the endothelial line automatically delineated by the system. The lower figure shows the marked movement trajectories of the anterior wall of the carotid artery, with red representing the movement towards the probe during systole and blue representing the movement away from the probe during diastole. On the right side, the measured values of PWVBS and PWVES, along with their standard deviations, are displayed.

### 2.4 Statistical methods

This study performed a retrospective analysis of the statistical data from the 56 participants using SPSS version 26.0 for statistical analysis. Measurement data were expressed as mean ± standard deviation ( $\bar{x}\pm \text{s}$). Data from both groups were verified via Q–Q plots to follow a normal distribution; therefore, independent-samples t-tests were used for between-group comparisons. Mann–Whitney U tests were used to compare subgroups with different UA levels within the overweight group. Scatterplots confirmed linear relationships between variables; thus, Pearson correlation analysis was used to assess the correlations of PWVBS and PWVES with IMT, BMI, FPG, UA, TG, TC, HDL-C, and LDL-C. Bland–Altman analysis was used to evaluate inter-operator agreement, and the intraclass correlation coefficient (ICC) was used to assess intra-operator repeatability. Receiver Operating Characteristic (ROC) curves were plotted to determine the cutoff values for PWVBS and PWVES in diagnosing impaired arterial elasticity. A significance level of *P*< 0.05 was considered statistically significant for all tests.

## 3 Results

### 3.1 Clinical data and biochemical data comparison between groups

The overweight group exhibited significantly higher levels of BMI, UA, TG, TC, HDL-C, and LDL-C compared to the normal group (*P*< 0.05). However, there was no statistically significant difference in age and FPG between the two groups (*P* > 0.05) (**Table 1**).

**Table 1:** Comparison of clinical and biochemical data between the two groups ( $\bar{x}\pm \text{s}$).

Variable	Normal group (n=26)	Overweight group (n=30)	*t*	*P*
Age (year)	13.42 ± 2.96	15.01 ± 3.01	0.66	0.079
BMI (Kg/m^2^)	18.65 ± 3.69	32.00 ± 4.76	0.432	<0.01
FPG (mmol/L)	4.89 ± 0.50	5.10 ± 0.58	0.418	0.158
UA(μmol/L)	335.19 ± 86.64	470.83 ± 90.61	0.013	<0.01
TG (mmol/L)	1.00 ± 0.61	1.72 ± 0.83	2.95	<0.01
TC (mmol/L)	4.12 ± 0.64	4.78 ± 0.53	0.74	<0.01
HDL-C(mmol/L)	1.36 ± 0.23	1.13 ± 0.14	3.06	<0.01
LDL-C(mmol/L)	2.51 ± 0.50	2.99 ± 0.46	0.166	<0.01

### 3.2 Comparison of IMT, PWVBS, and PWVES between groups

**Figure 3:**
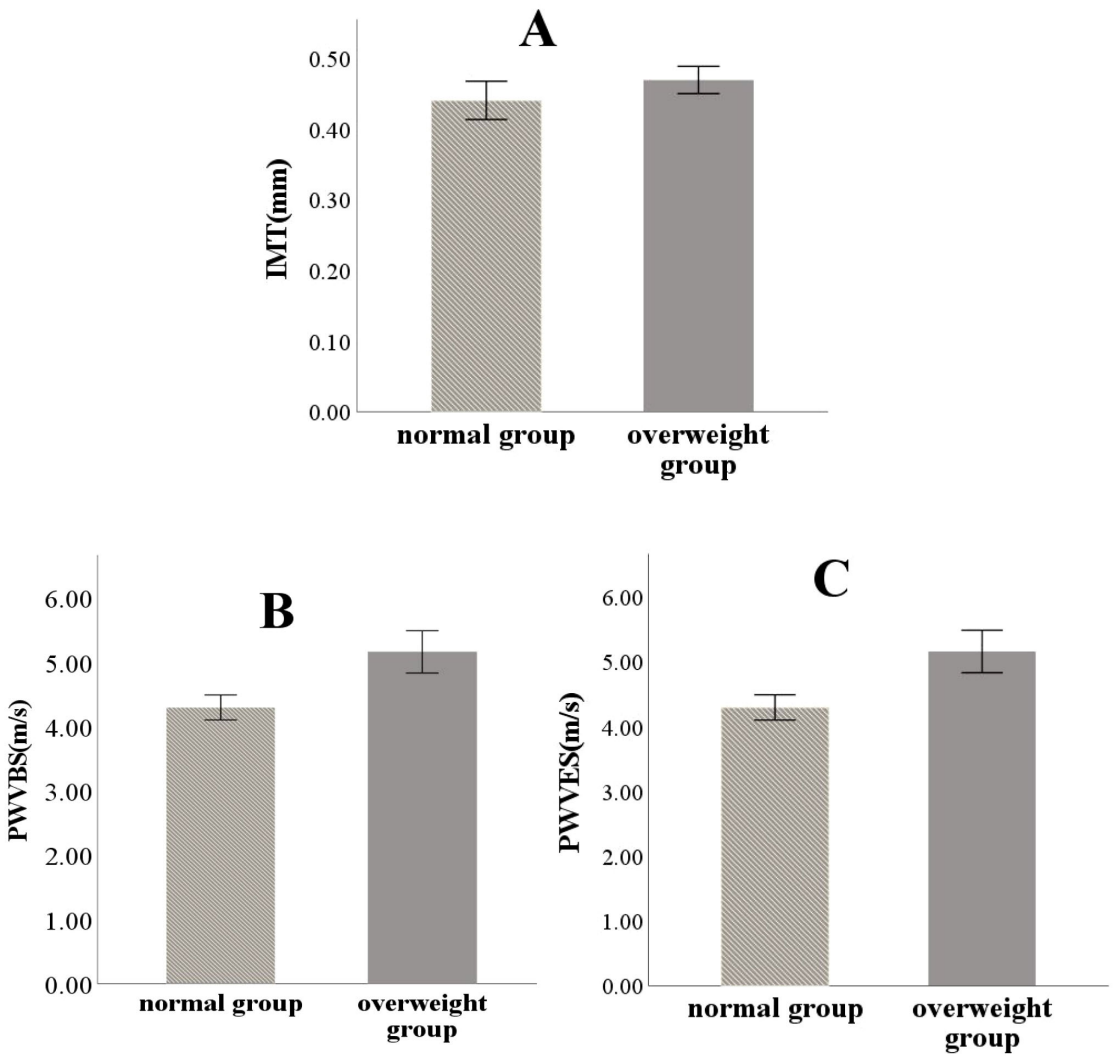
Comparison of IMT **(A)**, PWVBS **(B)** and PWVES **(C)** between the two groups.

The average values of IMT, PWVBS, and PWVES in the overweight group were all higher than those in the normal group. Among these, the differences in PWVBS and PWVES were statistically significant (*P*< 0.05), while no significant difference was observed in IMT (**Table 2** and **Figure 3**).

**Table 2:** Comparison of IMT, PWVBS and PWVES between the two groups ( $\bar{x}\pm \text{s}$).

Variable	Normal group (n=26)	Overweight group (n=30)	*t*	*P*
IMT(mm)	0.44 ± 0.07	0.47 ± 0.05	0.54	0.08
PWVBS(m/s)	4.30 ± 0.48	5.16 ± 0.88	3.89	<0.01
PWVES(m/s)	4.42 ± 0.39	5.25 ± 0.84	11.67	<0.01

**Table 3:** Comparison of carotid artery parameters between different UA level subgroups in overweight group ( $\bar{x}\pm \text{s}$).

Variable	Normal UA group (n=7)	Elevated UA group (n=23)	*U*	*Z*	*P*
IMT (mm)	0.45 ± 0.04	0.47 ± 0.05	91.0	0.519	0.604
PWVBS (m/s)	4.34 ± 0.35	5.25 ± 0.95	121.5	2.001	0.044
PWVES (m/s)	4.48 ± 0.70	5.34 ± 0.92	119.5	1.913	0.049

**Figure 4:**
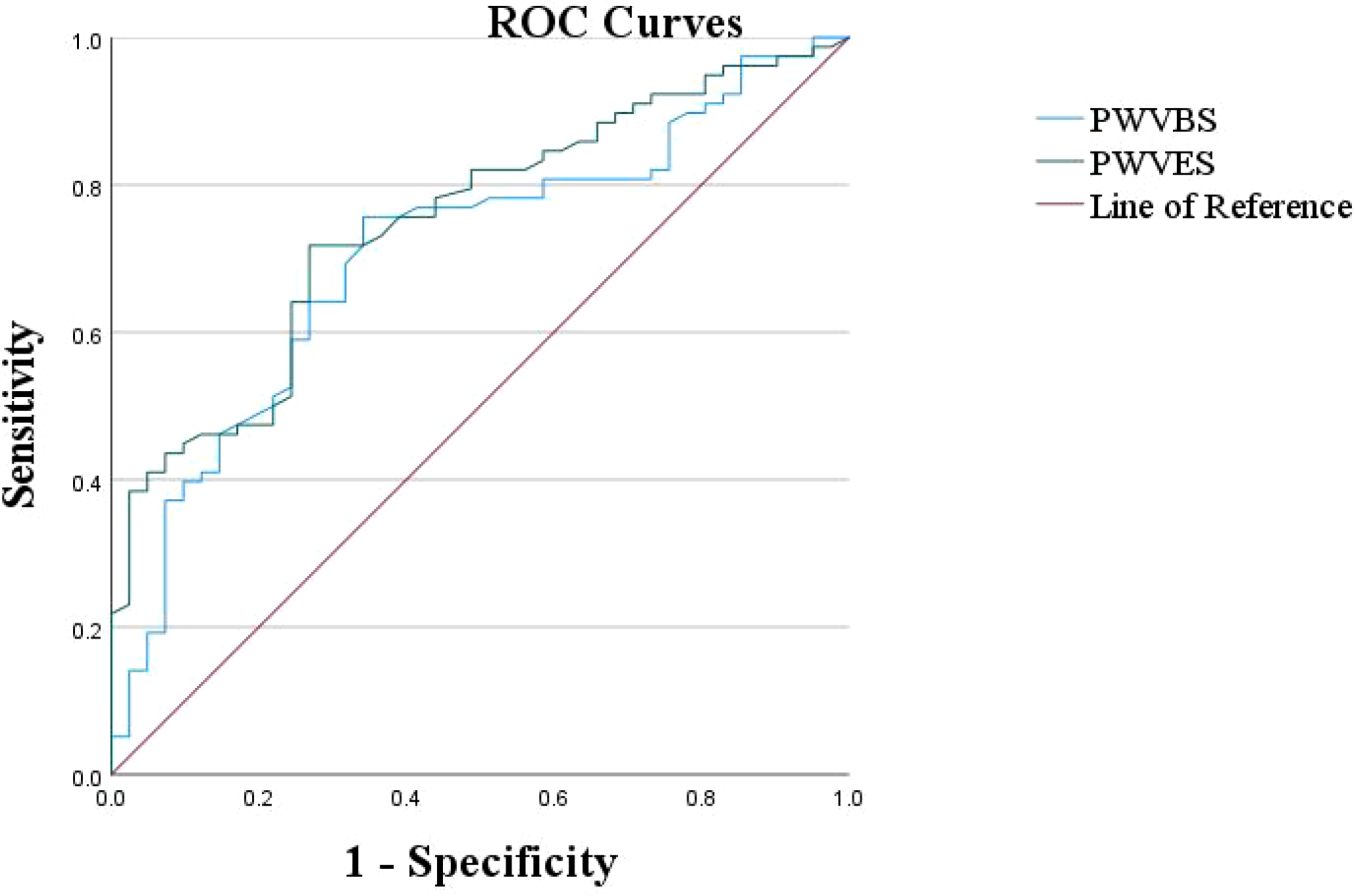
ROC curves of PWVBS and PWVES.

### 3.3 Comparison of carotid parameters among different UA levels in the overweight group

According to the normal uric acid values of 155-357μmol/L established by our hospital, the overweight group was divided into two subgroups: the elevated UA group (UA > 357μmol/L) with 23 cases and the normal UA group (UA≤ 357μmol/L) with 7 cases. The Mann–Whitney U test showed that PWVES differed significantly between the two groups (*P* < 0.05, *r* = 0.35), with a 95% CI for the median difference of [5.03, 5.77].PWVBS also differed significantly (*P* < 0.05, *r* = 0.37), with a 95% CI for the median difference of [4.74, 5.58]. No significant difference was found in IMT (*P* > 0.05) (**Table 3**).

### 3.4 Correlation of PWVBS and PWVES with clinical data

In the overweight group, both PWVBS and PWVES exhibited positive correlations with BMI, UA, TC, and LDL-C, while negative correlations were observed with HDL-C (**Table 4**).

**Table 4:** The correlation between PWVBS, PWVES and various clinical factors was analyzed.

Variable		PWVBS(m/s)		PWVES(m/s)	
		*r*	*P*	*r*	*P*
BMI(Kg/m^2^)		0.54	<0.05	0.54	<0.05
UA(μmol/L)		0.52	<0.05	0.44	<0.05
TC (mmol/L)		0.34	<0.05	0.32	<0.05
LDL-C (mmol/L)		0.33	<0.05	0.33	<0.05
HDL-C (mmol/L)		-0.43	<0.05	-0.42	<0.05

### 3.5 Correlation of PWVBS and PWVES with clinical data

Assessment of agreement and repeatability of PWVBS and PWVES using UFPWV technology.

#### 3.5.1 Agreement assessment

Two operators each performed three consecutive measurements of carotid PWVBS and PWVES in the same participant and recorded the mean values. Bland–Altman plots were constructed to evaluate inter-operator agreement. The 95% limits of agreement for both PWVBS and PWVES were narrow, with only 3 out of all data points falling outside the 95% LoA—deemed clinically acceptable. These results indicate good agreement between the two operators.

#### 3.5.2 Repeatability assessment

Repeatability of PWVBS and PWVES measurements by the two operators using UFPWV was excellent, with intraclass correlation coefficients (ICCs) of 0.88 and 0.81, respectively.

### 3.6 Sensitivity and specificity analysis of PWVBS and PWVES

Using BMI as a status variable, ROC curves were plotted based on the carotid elasticity parameters PWVBS and PWVES. The areas under the curve (AUC) were found to be 0.707 for PWVBS and 0.749 for PWVES, indicating that PWVES has superior diagnostic efficacy for overweight. The optimal cutoff values were determined based on the maximum Youden index. When PWVBS was set at 4.33 m/s, the sensitivity and specificity were optimal, with sensitivity at 75.6% and specificity at 65.9%. For PWVES, at 4.515 m/s, the optimal sensitivity and specificity were found to be 71.8% and 73.2%, respectively. Therefore, this study considers PWVBS > 4.33 m/s and PWVES > 4.515 m/s as critical values for detecting changes in carotid elasticity among overweight adolescents (**Figure 4**).

## 4 Discussion

Overweight is a traditional risk factor for AS, which serves as the fundamental pathological basis for CVD. As of 2023, the global prevalence of CVD is estimated at 775 million, making it a leading cause of health loss worldwide, responsible for approximately two-thirds of all health losses (18). Therefore, monitoring overweight and carotid elasticity impairment during adolescence could effectively delay the progression of AS, thereby reducing the prevalence of CVD in adulthood.

This study jointly analyzed the association between clinical variables (e.g., age and sex) and arterial elasticity parameters in adolescents. No statistically significant difference in age was observed between the two groups, and age showed no correlation with elasticity parameters, suggesting that age has minimal impact on carotid arterial elasticity in overweight adolescents. Age is a well-established factor influencing arterial elasticity, with vascular elasticity typically declining with advancing age. This age-related decline is primarily manifested as structural alterations in the arterial wall—including elastin fragmentation and degeneration, along with increased collagen deposition—leading to reduced arterial compliance. However, since all participants in this study were adolescents and middle-aged or older individuals were excluded, the narrow and homogeneous age distribution of the sample may explain the absence of a significant association between age and arterial elasticity parameters. Furthermore, this study found that sex did not significantly affect vascular elasticity. Although some studies have reported that estrogen in females exerts anti-atherosclerotic effects (19), and that excess androgens can impair vascular endothelial function (20) and promote vascular smooth muscle cell proliferation and migration—thereby increasing arterial stiffness—the participants in this study were adolescents, who generally have lower levels of sex hormone secretion compared to adults. This may partly explain the lack of significant differences in arterial elasticity between sexes in our cohort.

The results of this study indicate that the carotid elasticity parameters PWVBS and PWVES in the overweight group were significantly higher than those in the normal group (*P*< 0.05), suggesting that being overweight leads to vascular wall damage and a decrease in vascular elasticity. A potential cause for this is the excessive accumulation of oxidized low-density lipoprotein cholesterol (ox-LDL-C) in the bodies of overweight individuals, which can damage the endothelium (21). Endothelial dysfunction is considered a critical early step in the pathophysiology of atherosclerosis. Endothelial cells play a pivotal role in regulating vascular tone by releasing various vasoactive factors, such as nitric oxide (NO), which maintains vasodilatory function. Abnormal deposition of ox-LDL-C in the arterial wall impairs endothelial permeability at an early stage, primarily manifesting as reduced release of vasoactive factors and diminished vasodilatory capacity, thereby adversely affecting vascular elasticity (22). Furthermore, ox-LDL-C disrupts the endothelial barrier, triggering oxidative stress and vascular inflammation. Sustained and excessive endoplasmic reticulum stress not only induces macrophage apoptosis but also promotes necrotic core formation in advanced atherosclerotic plaques—processes that are central to the initiation and progression of atherosclerosis (23). In addition, insulin resistance commonly present in overweight adolescents can directly induce hypertrophy and phenotypic modulation of both endothelial and vascular smooth muscle cells, leading to endothelial dysfunction and arterial stiffening (24). Notably, this study found no significant difference in IMT between the two groups, suggesting that structural changes in the carotid artery lag behind functional alterations in overweight adolescents.

In this study, the overweight group was further subdivided based on our hospital’s normal UA levels (155-357μmol/L), creating a UA normal subgroup (UA≤ 357μmol/L) and a UA elevated subgroup (UA > 357μmol/L). Comparison of carotid IMT and elasticity parameters revealed that the PWVBS and PWVES in the elevated UA group were significantly higher than those in the normal UA group (*P*< 0.05), with no statistical difference in IMT (*P* > 0.05). These findings suggest that elevated UA levels in overweight adolescents may further impair vascular elasticity. Furthermore, our analysis showed that UA had positive correlations with PWVBS and PWVES, indicating that UA may accelerate the progression of AS in overweight adolescents. Possible mechanisms include the following: First, visceral fat in overweight adolescents, which induces an influx of free fatty acids into the liver and hepatic portal vein, stimulating TG synthesis and leading to excessive production of UA via activated UA synthesis pathways (25). Elevated UA levels can cause endothelial dysfunction and promote the oxidation of LDL-C and lipid peroxidation (26). Oxidative stress responses within the vast majority of cells activate sterol regulatory element-binding proteins, resulting in the production of various lipogenic enzymes. All these lipases promote the production of fat (27). Second, insulin resistance may be the underlying link connecting hyperuricemia and hyperlipidemia (28). When both hyperlipidemia and hyperuricemia coexist, their combined risk is not merely additive; rather, they interact synergistically through mechanisms such as inflammation, oxidative stress, endothelial injury, and insulin resistance. This synergistic effect is particularly evident in arterial function assessed by UFPWV, substantially amplifying the risk of AS beyond that posed by either risk factor alone. Notably, no significant difference in IMT was observed between the groups, indicating that even in the presence of these two AS risk factors, structural alterations in the arterial wall are not yet detectable in adolescents. This further supports the notion that IMT is insufficiently sensitive to reflect early atherosclerotic changes in this population.

Additionally, this study analyzed the correlation of several common biochemical indicators with elasticity parameters, including UA, TC, LDL-C, and HDL-C. Elasticity parameters were positively correlated with TC, LDL-C, and UA, while a negative correlation was observed with HDL-C. LDL-C is well-known as a key factor in AS, as its oxidized products (ox-LDL-C) cannot be recognized by normal LDL receptors but are rather taken up by scavenger receptors on macrophages, promoting foam cell formation and eventually leading to early AS lesions known as fatty streaks. Conversely, HDL-C is currently regarded as an important player in counteracting AS (29). Its reverse cholesterol transport mechanism effectively reduces the abnormal accumulation of cholesterol in the vascular endothelium, thereby protecting endothelial cells and maintaining vascular elasticity. Moreover, triglyceride-rich lipoproteins (like intermediate-density lipoproteins, very-low-density lipoproteins, and chylomicron remnants) can directly or indirectly affect the structure of LDL-C and HDL-C (23), contributing to the formation of AS plaques. UA, as the final product of purine metabolism catalyzed by xanthine oxidase, generates reactive oxygen species, such as superoxide anions, during its synthesis. These free radicals can react with nitric oxide, damaging endothelial function. A study has confirmed that xanthine oxidase may serve as an important therapeutic target for the prevention and treatment of CVD (30). Therefore, early and effective monitoring of arterial elasticity changes and lipid and uric acid levels in overweight adolescents can help reduce the future risk of CVD.

Through ROC curve analysis, it was found that the AUC for the carotid elasticity parameters PWVBS and PWVES were 0.707 and 0.749, respectively. The optimal cutoff values determined using the Youden index indicated that PWVBS > 4.33 m/s and PWVES > 4.515 m/s could serve as diagnostic thresholds for abnormal carotid elasticity in overweight adolescents. Notably, the predictive value of PWVES (AUC = 0.749) was higher than that of PWVBS (AUC = 0.707), suggesting that PWVES can more effectively identify early changes in arterial elasticity among overweight adolescents, a finding consistent with previous research (31).

However, this study also has some limitations: (1) Perhaps the issue of adolescent overweight has not received sufficient attention, and the exclusion criteria are rather strict, which has resulted in a relatively small sample size for this study. In the future, it is necessary to increase publicity or conduct free screenings in schools to expand the sample size and scope; (2) The current study used BMI as a standard for classifying overweight status, but it has been reported that there are substantial individual differences in the correlation between BMI and both subcutaneous and visceral fat (32). Therefore, subsequent analyses should incorporate other obesity assessment parameters, such as waist circumference, waist-to-hip ratio, body fat percentage, and visceral fat area for a more comprehensive evaluation. (3) Due to the limited sample size, propensity score matching(PSM) was not employed, as matching could further reduce the effective sample size and increase estimation variance. Future studies with larger samples should incorporate PSM or multivariable regression models to adequately control for confounding factors (4). Although we adjusted for known covariates as thoroughly as possible in our analyses, residual confounding may still exist—particularly from unmeasured or imperfectly adjusted variables such as pubertal stage (e.g., Tanner staging) and socioeconomic status, which could potentially influence the primary outcomes. Future research should validate our findings in larger, multicenter cohorts.

## 5 Conclusion

In summary, this study innovatively employed UFPWV technology to assess carotid elasticity in overweight adolescents, revealing that the decline in arterial elasticity occurs earlier than morphological changes in the carotid artery. In the past, UFPWV technology was often used to evaluate the arterial elasticity of metabolic diseases in adults, but few studies were conducted in adolescents. However, in fact, the occurrence and development of AS in adults often originates from adolescents. Therefore, adolescents as the primary screening object can effectively prevent the incidence of CVD and other diseases in adults. The findings of this study indicate that UFPWV technology can facilitate precise evaluations of carotid vascular wall elasticity. Qualitative and quantitative analyses of carotid elasticity using UFPWV technology can aid in the early identification of vascular functional abnormalities in overweight adolescents, thereby providing timely interventions to reduce the risk of CVD in later life.

## Data Availability

The raw data supporting the conclusions of this article will be made available by the authors, without undue reservation.

## References

[1] The GBD 2015 Obesity Collaborators Afshin A Forouzanfar MH Reitsma MB . Health effects of overweight and obesity in 195 countries over 25 years. N Engl J Med. (2017) 377:13–27. doi: 10.1056/NEJMoa1614362, PMID: 28604169 PMC5477817

[2] Zhang X Liu J Ni Y Yi C Fang Y Ning Q . Global prevalence of overweight and obesity in children and adolescents. JAMA Pediatr. (2024) 178:800–13. doi: 10.1001/jamapediatrics.2024.1576, PMID: 38856986 PMC11165417

[3] Hiba J Aaron S Grace O Baur L . Obesity in children and adolescents: epidemiology, causes, assessment, and management. Lancet Diabetes Endocrinol. (2022) 10:351–65. doi: 10.1016/S2213-8587(22)00047-X, PMID: 35248172 PMC9831747

[4] Timothy Garvey W Mechanick JI . Proposal for a scientifically correct and medically actionable disease classification system (ICD) for obesity. Obesity. (2020) 28:484–92. doi: 10.1002/oby.22727, PMID: 32090513 PMC7045990

[5] Močnik M Varda Nataša Marčun . Lipid biomarkers and atherosclerosis—Old and new in cardiovascular risk in childhood. Int J Mol Sci. (2023) 24:2237. 10.3390/ijms24032237 36768558 PMC9916711

[6] Wu F David RJ Stephen RD Kähönen M Woo J Sinaiko A . Non–high-density lipoprotein cholesterol levels from childhood to adulthood and cardiovascular disease events. JAMA. (2024) 331:1834–44. doi: 10.1001/jama.2024.4819, PMID: 38607340 PMC11151142

[7] Antonio B Gordana IC Filip M Pfeifer L Bulum M Divjak E . Contribution of ultraFast™ Ultrasound and shear wave elastography in the imaging of carotid artery disease. Diagnostics. (2022) 12:1168. 10.3390/diagnostics12051168 35626326 PMC9140890

[8] Wang Y Zhao C Meng P Yu Y Li G Kong F . Incremental value of carotid elasticity modulus using shear wave elastography for identifying coronary artery disease in patients without carotid plaque. J Hypertens. (2020) 07:23–30. doi: 10.1097/HJH.0000000000002773, PMID: 33323910

[9] Mubarak RM Swarnalakshmi S Ruthira Eshanth VN Vignesh M . Evaluation of carotid intima-medial thickness by B’mode ultrasonography in hypertensive patients compared with normotensive patients. Int J Acad Med Pharm. (2023) 5:29–33. doi: 10.47009/jamp.2023.5.5.6

[10] Ejiri K Ding N Kim E Cainzos-Achirica M Tanaka H Howard-Claudio C . Association of segment-specific pulse wave velocity with vascular calcification: the ARIC (Atherosclerosis risk in communities) study. J Am Heart Assoc. (2024) 13:e031778. doi: 10.1161/JAHA.123.031778, PMID: 38214278 PMC10926832

[11] An X Li YH Shi SS Ge LL Li YH . Clinical significance and influencing factors of carotid pulse wave velocity in patients with diabetic microangiopathy. Clin Ultrasound. (2022) 50:309–16. doi: 10.1002/jcu.23153, PMID: 35150445

[12] Bai X Liu WJ Huang H You H . Logistic regression model based on ultrafast pulse wave velocity and different feature selection methods to predict the risk of hypertension. Public Health. (2022) 51:2099–107. Available online at: https://creativecommons.org/licenses/by-nc/4.0/. 10.18502/ijph.v51i9.10565 PMC9884380 36743358

[13] Li Y Zhang J An X Li Y . Evaluation of carotid artery elastic function using ultrafast pulse wave velocity and related influential factors in patients with rheumatoid arthritis. Echocardiography. (2022) 39:552–60. doi: 10.1111/echo.15325, PMID: 35212028

[14] Li ZJ Liu Y Du LF Luo XH . Evaluating arterial stiffness in type 2 diabetes patients using ultrasonic radiofrequency. J Huazhong Univ Sci Technolog Med Sci. (2016) 36:442–8. doi: 10.1007/s11596-016-1606-7, PMID: 27376818

[15] Yin LX Ma CY Wang S Wang Y Meng P Pan X . Reference values of carotid ultrafastPulse-wave velocity: A prospective,Multicenter, population-based study. J Am Soc Echocardiography. (2021) 6:629–41. doi: 10.1016/j.echo.2021.01.003, PMID: 33422666

[16] Gong Q Xue N . Application and progress of ultrasound technology in atherosclerosis. Adv Ultrasound Diagn Ther. (2023) 7:8–15. doi: 10.37015/AUDT.2023.220030

[17] National Health Commission of the PRC . Screening for overweight and obesity among school-age children and adolescents. WS/T 586-2018. (Beijing: Standards Press of China). (2018).

[18] Mensah GA Fuster V Murray CJL Roth GA . Global burden of cardiovascular diseases and risks, 1990-2022. J Am Coll Cardiol. (2023) 82:2350–473. doi: 10.1016/j.jacc.2023.11.007, PMID: 38092509 PMC7615984

[19] Zhu Z Chen L Jiang X Wu Y Zou C Luan Y . Absent atherosclerotic risk factors are associated with carotid stiffening quantified with ultrafast ultrasound imaging. Eur Radiol. (2014) 7):125–34. 10.1007/s00330-020-07405-4 33068187

[20] Hu Y Chen B Pan Y Xing K Xiao Z Sheng B . Evaluation of carotid artery elasticity and its influencing factors in non-obese PCOS patients using a technique for quantitative vascular elasticity measurement. Front Endocrinol. (2010) 7):132–8. doi: 10.3389/fendo.2024.1374718, PMID: 39314523 PMC11416955

[21] Shan H Guo D Zhang S Qi H Liu S Du Y . SNHG6 modulates oxidized low-density lipoprotein-induced endothelial cells injury through miR-135a-5p/ROCK in atherosclerosis. Cell Biosci. (2020) 10:4. doi: 10.1186/s13578-019-0371-2, PMID: 31921409 PMC6947907

[22] Liu WJ Chen ZS Dakota O Liu XB Huang XQ Wang LL . Arterial elasticity, endothelial function and intracranial vascular health: A multimodal MRI study. J Cereb Blood Flow& Metab. (2020), 1–8. doi: 10.1177/0271678X20956950, PMID: 33081567 PMC8142128

[23] Khatana C Neeraj K Saini SC Saini V Sharma A Sain R . Mechanistic insights into the oxidized low-density lipoproteinInduced atherosclerosis. Oxid Med Cell Longevity. (2020) 14:5245308. 10.1155/2020/5245308 PMC7512065 33014272

[24] Duca L Blaise S Romier B Laffargue M Gayral S El Btaouri H . Under pathological conditions, vascular and inflammatory cells can however produce tropoelastin, but these tropoelastin molecules fail to cross-link into mature elastic fibers. Matrix ageing and vascular impacts: focus on elastin fragmentation. Cardiovasc Res. (2016) 110:298–308. doi: 10.1093/cvr/cvw061, PMID: 27009176

[25] Zeng J Lawrence WR Yang J Tian J Li C Lian W . Association between serum uric acid and obesity in Chinese adults: a 9-year longitudinal data analysis. BMJ Open. (2021) 11:e041919. doi: 10.1136/bmjopen-2020-041919, PMID: 33550245 PMC7908911

[26] She D Xu W Liu Serum J Zhang Z Fang P Li R . Uric acid to creatinine ratio and risk of metabolic syndrome in patients with overweight/obesity. Diabetes Metab Syndr Obes. (2023) 16:3007–17. doi: 10.2147/DMSO.S427070, PMID: 37790260 PMC10544178

[27] Si SA Chen MQ Zhang GJ . Association of serum uric acid with hypertriglyceridemia in children and adolescents: a cross-sectional study. Lipids Health Dis. (2024) 23:195. doi: 10.1186/s12944-024-02182-1, PMID: 38915087 PMC11194951

[28] Krzystek-Korpacka M Patryn E Kustrzeba-Wojcicka I Chrzanowska J Gamian A Noczynska A . Gender-specific association of serum uric acid with metabolic syndrome and its components in juvenile obesity. Clin Chem Lab Med. (2011) 49:129–36. doi: 10.1515/CCLM.2011.011, PMID: 20961193

[29] Feingold KR . Introduction to lipids and lipoproteins. In: Feingold KR Anawalt B , editors. Comprehensive endocrinology book; endotext. MDText.com, Inc, South Dartmouth, MA, USA (2021).

[30] Sekizuka H . Uric acid, xanthine oxidase, and vascular damage: potential of xanthine oxidoreductase inhibitors to prevent cardiovascular diseases. Hypertens Res. (2022) 45:772–4. doi: 10.1038/s41440-022-00891-7, PMID: 35301451

[31] Liu B Si X Shan Y Xia W Li M Ge D . Clinical application value of Ultrafast Pulse Wave Velocity in early cardiovascular injury of Immunoglobulin A nephropathy. Med Ultrason. (2023), 1–6. doi: 10.11152/mu-4319, PMID: 38244220

[32] Potter AW Chin GC Looney DP Friedl K . Defining overweight and obesity by percent body fat instead of body mass index. J Clin Endocrinol Metab. (2025) 110:e1103–7. doi: 10.1210/clinem/dgae341, PMID: 38747476 PMC11913102

